# Efficacy of imidacloprid + moxidectin and selamectin topical solutions against the KS1 *Ctenocephalides felis *flea strain infesting cats

**DOI:** 10.1186/1756-3305-4-174

**Published:** 2011-09-13

**Authors:** Michael W Dryden, Patricia A Payne, Vicki Smith, Joe Hostetler

**Affiliations:** 1 Dept. of Diagnostic Medicine/Pathobiology Kansas State University Manhattan KS 66506, USA; 2 Bayer HealthCare, Animal Health PO Box 390 Shawnee Mission, KS 66201, USA

## Abstract

**Background:**

Two studies were conducted to evaluate and compare the efficacy of imidacloprid + moxidectin and selamectin topical solutions against the KS1 flea strain infesting cats. In both studies the treatment groups were comprised of non-treated controls, 6% w/v selamectin (Revolution^®^; Pfizer Animal Health) topical solution and 10% w/v imidacloprid + 1% w/v moxidectin (Advantage *Multi*^® ^for Cats, Bayer Animal Health) topical solution. All cats were infested with 100 fleas on Days -2, 7, 14, 21, and 28. The difference in the studies was that in study #1 efficacy evaluations were conducted at 24 and 48 hours post-treatment or post-infestation, and in study #2 evaluations were conducted at 12 and 24 hours.

**Results:**

In study #1 imidacloprid + moxidectin and the selamectin formulation provided 99.8% and 99.0% efficacy at 24 hours post-treatment. On day 28, the 24 hour efficacy of the selamectin formulation dropped to 87.1%, whereas the imidacloprid + moxidectin formulation provided 98.9% efficacy. At the 48 hour assessments following the 28 day infestations, efficacy of the imidacloprid + moxidectin and selamectin formulations was 96.8% and 98.3% respectively. In study # 2 the efficacy of the imidacloprid + moxidectin and selamectin formulations 12 hours after treatment was 100% and 69.4%, respectively. On day 28, efficacy of the imidacloprid + moxidectin and selamectin formulations 12 hours after infestation was 90.2% and 57.3%, respectively. In study #2 both formulations provided high levels of efficacy at the 24 hour post-infestation assessments, with selamectin and imidacloprid + moxidectin providing 95.3% and 97.5% efficacy, following infestations on day 28.

**Conclusions:**

At the 24 and 48 hour residual efficacy assessments, the imidacloprid + moxidectin and selamectin formulations were similarly highly efficacious. However, the imidacloprid + moxidectin formulation provided a significantly higher rate of flea kill against the KS1 flea strain infesting cats at every 12 hour post-infestation residual efficacy assessment. Both formulations should provide excellent flea control for an entire month on cats.

## Background

Numerous animals that travel through neighborhoods, parks and yards carry *Ctenocephalides felis *(cat fleas), including feral cats and wildlife, such as opossums, raccoons, foxes, and coyotes [1]. These flea-infested animals are continuously depositing flea eggs in the outdoor environment. Within a few weeks, eggs deposited in protected areas may develop into adult fleas. If pets come in contact with these areas, they can rapidly acquire fleas. Once on a pet, the fleas will feed and mate, after which female fleas will begin laying eggs within as little as 24 hours [2]. After a few days, each female flea will produce 40 to 50 eggs per day, with hundreds and potentially thousands of eggs being deposited back in the home [3].

Most pet owners never see the first two or three fleas their pets acquire and pets often go without flea preventive treatment for several days or weeks [4]. During that time, flea eggs are continually deposited in the home, with development of larvae, pupae, and eventually emerging adult fleas. At some point, there are enough fleas emerging within the home and on the pet that the problem becomes noticeable by the owner. But by that time, the home is already infested with hundreds to thousands of immature flea life stages that develop and emerge as adults to continually reinfest the pet [4,5].

When the flea infested pet is presented to a veterinarian, the resident flea population on the pet must be eliminated rapidly, but control of the infestation is ultimately achieved by eliminating the existing immature and mature flea life stages within the premises. As fleas emerge and jump on pets, it is critical to kill them as rapidly as possible. The more rapidly a residual flea product can kill newly acquired fleas, the more effectively it can manage flea allergy dermatitis, the more likely it can decrease the transmission of vector borne diseases, and fewer fleas are observed by pet owners [6]. An added benefit of an effective residual flea product is its contribution to reproductive suppression, attempting to kill newly acquired fleas before they can reach reproductive status [6].

The purpose of the two studies described in this article was to evaluate the initial and residual speed of kill of an imidacloprid + moxidectin topical formulation against the KS1 *Ctenocephalides felis*, flea strain infesting cats. The imidacloprid + moxidectin formulation was compared against a topical selamectin formulation, which had previously been evaluated against the KS1 cat flea strain. The KS1 cat flea strain has been maintained as a closed colony at Kansas State University since 1990. Previous studies have indicated that the KS1 strain has some level of resistance or reduced susceptibility to carbaryl, chlorpyriphos, fenthion, fipronil, imidacloprid, permethrin, pyrethrins, and spinosad [6-13].

## Methods

The two studies conducted in these investigations had overall similar study designs. The primary difference between the studies was that in study #1 speed of kill was evaluated at 24 and 48 hours post-treatment or post-infestation. Whereas in study #2, speed of kill was assessed at 12 and 24 hours post-treatment or post-infestation.

### Animals and housing

Study one (1) included the use of 34 purpose bred Domestic Short Hair cats (17 m:17 f) and study two (2) used 56 (28 m:28 f) purpose bred DSH cats between 7 and 12 months of age. The cats were housed in standard stainless steel cages. No drugs, baths, shampoos, or pesticides were administered to the cats during the preconditioning phase or during the course of the study, other than what was described in the protocol. All animal care procedures conformed to guidelines established by the Institutional Animal Care and Use Committee at Kansas State University (IACUC # 2833 & #2904).

### Animal Selection and Randomization

On day -7, cats in each study were infested with 100 cat fleas, *C. felis*, (KS1 strain) 1 to 5 days post emergence. On day -5, flea comb counts were performed to assess the ability of cats to maintain infestations. Cats were combed with a fine-toothed flea comb having 12-13 teeth/cm. Flea removal was achieved by combing each cat thoroughly for 10 min. If five or more fleas were recovered during this period, the cat was combed for an additional 5 min. If any fleas were recovered during the second combing period, the cats were combed for an additional 5 min.

In study #1 the 15 male cats and 15 female cats retaining the highest flea levels were retained for the study. In study #2 the 24 male cats and 24 female cats retaining the highest flea levels were retained for the study. Within each gender the cats were ranked in descending order by flea count. Cats were randomly grouped into replicates of three based on descending flea counts and allocated into one of three treatment groups (Study # 1-10 cats; 5 m/5 f: Study # 2-16 cats 8 m/8 f). Each group of cats was then randomly divided into two subgroups of 5 cats (Study #1) or 8 cats (Study #2) each. While cats were not allocated according to weight, the cats in the treatment groups were similar in size. In study #1 the cats in the three treatment groups weighed on average 3.27, 3.47 and 3.41 kg. While in study # 2 the cats in the three treatment groups weighed on average 3.31, 3.22 and 3.24 kg.

### Treatments

In each study, treatment groups were comprised of non-treated controls, 6%w/v selamectin (Revolution^®^; Pfizer Animal Health) topical solution, and 10% w/v imidacloprid + 1.0% w/v moxidectin (Advantage *Multi*^® ^for Cats, Bayer Animal Health) topical solution. In both studies the products were applied according to label directions.

### Efficacy Evaluations

To evaluate efficacy of the formulations in eliminating an existing flea infestation, all cats were infested with 100 fleas on Day -2 and treatments were applied on Day 0. In study #1, efficacy was determined by removing fleas from 5 cats in each treatment group at 24 hours, and 5 cats at 48 hours post-treatment. In study #2, efficacy was determined by removing fleas from 8 cats in each treatment group at 12 hours, and from 8 cats at 24 hours post-treatment. Residual activity was determined by reinfesting cats with 100 adult fleas on days 7, 14, 21 and 28 post-treatment and then removing live fleas in study # 1 from 5 cats in each treatment group at 24 hours and 5 cats at 48 hours post-reinfestation. In study #2 live fleas were removed from 8 cats in each treatment group at 12 hours and 8 cats at 24 hours post-reinfestation. Fleas were removed using the previously described flea combing procedure.

### Data analysis

For both studies, geometric means were calculated following transformation using a logarithmic method (averaging the transformed values, and converting the average using antilog to represent a geometric mean). All counts were modified by adding one (1) to each prior to logarithmic transformation and subtracting one (1) from the antilog value to meaningfully represent the geometric mean for each group.

Log (counts+1) were analyzed with an analysis of covariance (ANCOVA) for each study day where flea counts were measured. The pre-treatment counts were used as a covariate. SAS PROC MIXED from SAS version 9.2 (SAS^® ^Institute, Cary, NC) was used for all analyses. Differences in least square means were determined between all pair-wise combinations of the three treatment groups. The fixed effect of treatment groups was evaluated at the alpha level of 0.05.

## Results

In study #1, the imidacloprid + moxidectin and the selamectin topical solutions provided 99.8% and 99.0% efficacy within 24 hours of treatment (Table 1). When cats were reinfested on day 14, both formulations provided ≥ 98.3% efficacy within 24 hours post-infestation. On day 28 the efficacy of the selamectin formulation 24 hours post infestation decreased slightly to 87.1% (Table 1) while the imidacloprid + moxidectin topical solution provided 98.9% control. At the 48 hour assessment following the 28 day infestations both formulations provided ≥ 96.8% efficacy (Table 1).

**Table 1:** STUDY #1: Geometric mean flea counts and percent efficacy relative to nontreated controls for cats treated with an 10% w/v imidacloprid + 1.0% w/v moxidectin topical solution or a selamectin (6% w/v) topical spot-on, 24 and 48 hours after treatment or infestation.

	Day 0		Day 7		Day 14		Day 21		Day 28	
**Treatment^1^**	**Mean # of fleas^2,3^**	**% efficacy^4^**	**Mean # of fleas**	**% efficacy**	**Mean # of fleas**	**% efficacy**	**Mean # of fleas**	**% efficacy**	**Mean # of fleas**	% efficacy

24 hours post-treatment or infestation										

Controls	79.2a		67.4a		64.1a		68.6a		52.6a	

Selamectin	0.8b	99.0	0.0b	100	1.1b	98.3	6.0b	91.2	6.8b	87.1

Imidacloprid-Moxidectin	0.1b	99.8	0.2b	99.6	0.0b	100	5.4b	92.1	0.6c	98.9

48 hours post-treatment or infestation										

Controls	72.7a		69.7a		53.5a		56.8a		52.9a	

Selamectin	0.0b	100	0.0b	100	0.0b	100	0.5b	99.1	0.9b	98.3

Imidacloprid-Moxidectin	0.0b	100	0.0b	100	0.1b	99.7	0.6b	99.0	1.7b	96.8

^1^Each of 5 cats in the control group received no treatment. Each of 5 cats in the 10% w/v imidacloprid + 1.0% w/v moxidectin or selamectin (6%w/v) treatment groups were administered the topical spot-ons, according to label directions on Day 0.

^2^Each cat was infested with 100 adult *Ctenocephalides felis *from the KS1 strain on days -2, 7, 14, 21 and 28.

^3^Geometric mean # of fleas recovered from cats per treatment group.

^4^% efficacy = ((geometric mean count control-geometric mean count treatment)/geometric mean count treatment) × 100

a, b, c geometric means within a column with unlike letter superscripts are significantly different (P < 0.05).

In study # 2 the imidacloprid + moxidectin topical solution provided 100% efficacy within 12 hours post-treatment (Table 2) where as the selamectin topical solution only provided 69.4% efficacy. Throughout the next 28 days, both formulations provided similar high levels of efficacy at the 24 hour post-infestation assessments, with selamectin and imidacloprid + moxidectin providing reductions of 95.3% and 97.5% efficacy at the Day 29 count, respectively (Table 2). However, there were significant differences (P < 0.05) in residual efficacy at the 12 hour post-infestation assessments at every time period, with the imidacloprid + moxidectin formulation providing a more rapid residual speed of kill (Table 2) when compared to the non-treated control group and selamectin treated cats. By day 28 post-treatment, the 12 hour post-infestation efficacy for selamectin was 57.3%, while the imidacloprid + moxidectin formulation provided a 90.2% reduction in flea populations.

**Table 2:** STUDY #2: Geometric mean flea counts and percent efficacy relative to nontreated controls for cats treated with an 10% w/v imidacloprid + 1.0% w/v moxidectin topical solution or a selamectin (6%w/v) topical spot-on, 12 and 24 hours after treatment or infestation.

	Day 0		Day 7		Day 14		Day 21		Day 28	
**Treatment^1^**	**Mean # of fleas^2,3^**	**% efficacy^4^**	**Mean # of fleas**	**% efficacy**	**Mean # of fleas**	**% efficacy**	**Mean # of fleas**	**% efficacy**	**Mean # of fleas**	**% efficacy**

12 hours post-treatment or infestation										

Controls	52.2a		62.2a		56.4a		47.5a		48.4a	

Selamectin	16.0b	69.4	0.6b	99.1	5.3b	90.6	18.6a	60.9	20.6a	57.3

Imidacloprid-Moxidectin	0.0c	100.0	0.0c	100.0	0.2c	99.7	0.7b	98.5	4.7b	90.2

24 hours post-treatment or infestation										

Controls	45.1a		56.3a		52.4a		47.1a		49.6a	

Selamectin	1.6b	96.4	0.0b	100.0	0.1b	99.7	0.5b	99.0	2.3b	95.3

Imidacloprid-Moxidectin	0.0c	100.0	0.0b	100.0	0.1b	99.8	0.0b	100.0	1.2b	97.5

^1^Each of 8 cats in the control group received no treatment. Each of 8 cats in the 10% w/v imidacloprid + 1.0% w/v moxidectin or selamectin (6%w/v) treatment groups were administered the topical spot-ons, according to label directions on Day 0.

^2^Each cat was infested with 100 adult *Ctenocephalides felis *from the KS1 strain on days -2, 7, 14, 21 and 28.

^3^Geometric mean # of fleas recovered from cats per treatment group.

^4^% efficacy = ((geometric mean count control -geometric mean count treatment)/geometric mean count treatment) × 100

a, b, c geometric means within a column with unlike letter superscripts are significantly different (P < 0.05).

There were no adverse events associated with treatments in either study.

## Discussion

These studies demonstrated that the imidacloprid + moxidectin topical solution provided rapid initial speed of kill against the KS1 flea strain, with 100% efficacy within 12 hours of treatment. However, the initial speed of kill of selamectin was not quite as rapid. The results for the initial speed of kill of selamectin in this investigation were similar to results from a 2005 laboratory study against the KS1 flea strain; in this current investigation the 12 hour post-treatment efficacy was 69.4% and the 12 hour post-treatment efficacy in the 2005 study was 59.7% [10]. It is unknown why selamectin has a slower initial speed of kill, but may be related to the systemic activity of the product and its need to be absorbed across the skin to reach effective blood levels.

In addition the residual efficacy of selamectin in this study against the KS1 flea strain was similar to the residual efficacy observed in the 2005 study [10]. In the current investigation the 12 hour post-infestation residual efficacy assessments efficacy ranged from 99.1% on day 7 to 57.3% on day 28. In the 2005 study, at the same post-treatment time points, the efficacy was 99.4% and 70.9%. Also in the 2005 study the efficacy observed when fleas were removed 48 hours after the 28 day infestation was 99.0%, [10] while in the current investigation the efficacy at the same time period was 98.3%. Additionally, the 24 hour residual efficacy of selamectin on day 28 in the 2005 study was 90.1%, while in the current investigation it was 87.1% in study #1 and 95.3% in study #2. Even though these studies were conducted over 5 years apart, the efficacy assessments for selamectin against the KS1 fleas strain were remarkably similar.

Also of interest is the difference in residual efficacy between the imidacloprid + moxidectin topical solution in this study and the 9.1% w/w imidacloprid formulation evaluated in the 2005 study. In the previous 2005 speed of kill study against the KS1 flea strain, the imidacloprid topical solution did not provide a more rapid residual speed of kill at 12 hours post-infestation at 7, 14, 21 or 28 days post-treatment than the selamectin formulation [10]. While, in this current investigation imidacloprid + moxidectin topical solution provided a more rapid residual speed of kill at every 12 hour post-infestation assessment. In 2005 the 12 hour post-infestation efficacy at 21 and 28 days for imidacloprid mono topical solution was 65.2% and 61.6%, respectively [10]. In this current investigation the 12 hour post-infestation efficacy at 21 and 28 days for imidacloprid + moxidectin was 98.5% and 90.2%, respectively.

While direct statistical comparison between the current and 2005 study cannot be conducted, it appears that the imidacloprid + moxidectin formulation provides a more rapid residual speed of kill against the KS1 flea strain than imidacloprid mono topical solution. Whether the more rapid residual speed of kill is from an additive or synergistic effect of the moxidectin is currently unknown.

It has previously been demonstrated that several flea products do not perform well against the KS1 flea strain either due to resistance or innate reduced susceptibility [6-13]. It has been demonstrated that the residual speed of kill of older pyrethroid and organophosphate based flea products is very poor against this flea strain due to resistance [6,8,11]. In addition more modern insecticides such as fipronil, imidacloprid, and spinosad also have reduced activity against the KS1 strain [7,9,10,12,13]. These newer insecticides were introduced into the U.S. as flea products 6 years (fipronil and imidacloprid) or 17 years (spinosad) after the KS1 strain was colonized. Various studies using other cat flea strains have reported that the 28-30 day residual efficacy of fipronil, imidacloprid, and spinosad flea products should range from approximately 95% to 100% [7,14-21]. However, when these formulations were evaluated against the KS1, strain the 28-30 day residual efficacy was markedly reduced [7,10,12,13]. While these insecticides have reduced residual activity against the KS1 strain, dinotefuran, metaflumizone and selamectin topical spot-on formulations have demonstrated excellent residual efficacy against this strain [10,12,13,22].

## Conclusions

Based on the current studies reported here, the imidacloprid + moxidectin combination topical formulation was highly effective against the KS1 flea strain, with rapid residual speed of kill, killing 90.2% of fleas within 12 hrs after infestation 28 days post-treatment, and should provide effective residual flea control on flea infested cats.

## Competing interests

MWD has served as a consultant and has been sponsored to lecture by Bayer Animal Health and Pfizer Animal Health, manufacturers of Revolution^® ^and Advantage *Multi*^® ^for Cats, products that were evaluated in these investigations. JH is a veterinarian employed by Bayer Animal Health and these studies were funded in part by and publications fees paid by Bayer Animal Health.

## Authors' contributions

MWD conceived, designed, supervised the study and drafted the paper. PAP assisted in design of study, data collection and revision of manuscript; VS coordinated and supervised data collection and entry and revision of manuscript; JH assisted in design of study, monitoring of study and manuscript revision. All authors reviewed and approved the final manuscript.
